# Costimulation blockade and Tregs in solid organ transplantation

**DOI:** 10.3389/fimmu.2022.969633

**Published:** 2022-09-02

**Authors:** Moritz Muckenhuber, Thomas Wekerle, Christoph Schwarz

**Affiliations:** ^1^ Division of Transplantation, Department of General Surgery, Medical University of Vienna, Vienna, Austria; ^2^ Division of Visceral Surgery, Department of General Surgery, Medical University of Vienna, Vienna, Austria

**Keywords:** Treg - regulatory T cell, transplantation, costimulation blockade, CTLA4 Ig, IL-2, immunosuppressant, costimulation

## Abstract

Regulatory T cells (Tregs) play a critical role in maintaining self-tolerance and in containing allo-immune responses in the context of transplantation. Recent advances yielded the approval of the first pharmaceutical costimulation blockers (abatacept and belatacept), with more of them in the pipeline. These costimulation blockers inhibit effector cells with high clinical efficacy to control disease activity, but might inadvertently also affect Tregs. Treg homeostasis is controlled by a complex network of costimulatory and coinhibitory signals, including CD28, the main target of abatacept/belatacept, and CTLA4, PD-1 and ICOS. This review shall give an overview on what effects the therapeutic manipulation of costimulation has on Treg function in transplantation.

## Introduction

### Tregs

Natural, thymus-derived regulatory T cells express CD4 and are characterized by the surface expression of the high affinity chain (alpha-unit) of the IL-2 receptor (CD25) and a diminished expression of the alpha-unit of the IL-7 receptor (CD127). They are further defined by the expression of the X-chromosome encoded transcription factor forkhead box P3 (FoxP3), which controls Treg development, plasticity and stability (1). Of note, FoxP3 is not absolutely required for a suppressive T cell phenotype. There are defined subsets of T cells which do not express FoxP3^-^ but have suppressive function (2, 3). Tr1cells are a prominent example of FoxP3- regulatory cells, with critical roles in suppression of inflammation (4) and with therapeutic potential (5). There are two main types of FoxP3+ Tregs that can be distinguished with overlapping features but also distinct properties. The main proportion of Tregs consists of thymic-derived Tregs (tTregs, formerly classified as natural Tregs (nTregs)). tTregs develop in the thymus and are typically characterized by the expression of helios (6) and neuropilin 1 (nrp1) (7, 8). In contrast to that, peripheral Tregs (pTregs) develop from CD4^+^ FoxP3^–^ cells upon antigen stimulation in the presence of distinct anti-inflammatory cytokines. pTregs develop primarily in the intestinal system (9) and the placenta (10). Whereas tTregs are supposed to be essential to control systemic and tissue specific autoimmunity, pTregs control commensal microbiota composition and Th2 responses (11).

Tregs are crucial to maintain self-tolerance and control an overall immune response (12). It has been shown, that absence or mutation of FoxP3 leads to severe autoimmune disease in mice (scurfy phenotype) (13, 14) and humans respectively (IPEX immune dysregulation, polyendocrinopathy, enteropathy, X-linked syndrome) (3, 15). In humans, approximately 1-3% of all CD4^+^ T cells are regulatory T cells (16, 17), however, the numbers may vary substantially between individuals, and further show a distinct distribution in various tissues in the human body. The pleiotropic mechanisms of action of Treg mediated immune modulation include the production of anti-inflammatory cytokines (IL-10, TGFβ, IL-35) (18), expression of co-inhibitory molecules (CTLA4, PD-1, LAG3) (19, 20) and cytotoxic suppression *via* granzyme A, B and perforin (18, 21). Additionally, IL-2 consumption *via* the high affinity unit of the IL-2 receptor (CD25) contributes to a down regulation of an overall immune response. Moreover, Tregs remove peptide-MHC complexes from the surface of dendritic cells (DC), thereby leading to antigen-specific regulation (22). Several of these mechanisms are often contributing to regulation. Notably, the expression of CTLA4 seems particularly important as Treg-specific CTLA4 deficiency results in an impaired *in vivo* and *in vitro* suppressive Treg function (20, 23). A main mechanism of CTLA4 is the removal of B7 molecules from the surface of antigen presenting cells (mainly migratory dendritic cells) by CTLA4-mediated trans-endocytosis (19, 24).

Several costimulation blockers have been approved for clinical use and many more are currently investigated in preclinical studies. Given the tight interplay between Tregs and costimulatory signals, knowledge about these interactions is crucial especially in diseases where Tregs play an important role (e.g. transplantation, auto-immune disease, cancer…).

### Tregs in transplantation

As a pivotal part in regulation of the immune system, Tregs also play a major role in allogeneic transplantation (25, 26). In this context, Tregs can intercept at several critical steps during allo-immune responses: Tregs can prevent priming of indirectly alloreactive T cells by removing peptide-MHC complexes and B7 molecules from the surface of dendritic cells. Furthermore, Tregs can restrict expansion of allo-antigen specific follicular T and B-cells and thereby confine humoral allo-immunity (27). Within the allograft itself, Tregs can create a privileged environment through consumption of IL-2 and secretion of immunosuppressive cytokines and metabolites like IL-10 and Adenosine (28). Through *infectious tolerance*, new generations of Tregs can be recruited to and induced within the allograft. Thereby Treg-mediated intra-graft regulation might be self-sustaining (29).

Accordingly, operationally tolerant patients with a liver allograft display significantly higher levels of Tregs than matched control patients (30, 31). Tregs were also shown to be indispensable for deliberate induction and maintenance of donor-specific transplantation tolerance in several models (32).

Given the important role in immune regulation, the exploitation of Tregs has become an attractive aim in order to reduce life-long immunosuppression. In preclinical studies the therapeutic use of Tregs prolongs allograft survival (33–35). Currently, the potential of adoptive Treg therapy in solid organ transplantation is explored in several clinical trials (36–39) with the first preliminary evidence emerging for the efficacy of Treg therapy (40).

### Homeostatic control of Tregs

Several factors contribute to Treg homeostasis to maintain numbers within a physiological range. One major stimulus is signaling *via* the IL-2 receptor and activation of the STAT5 signaling pathway. As Tregs are incapable of self-producing IL-2, abundance of this cytokine is crucial for Tregs survival especially in mature FoxP3 positive regulatory T cells (41, 42). Interestingly, FoxP3 induces a pro-apoptotic protein signature and a reduced expression of pro survival Bcl-2 molecules, leading to FoxP3 induced death in most newly arising Tregs. This lethality can be prevented in presence of (the limited) IL-2 signaling *via* the common gamma chain (43). As Tregs consume IL-2, Treg depletion leads to higher levels of IL-2 underlying the importance of Tregs in controlling the abundance of IL-2 (44).

However, Treg homeostasis and function is tightly regulated *via* numerous costimulatory signals in order to keep the fine balance between immunosuppression (potentially resulting in infection or malignancy) and avoiding excessive immune activation and autoimmunity.

## The complex crosstalk between PD1-PDL1 and CD28/CTLA4-B7

On conventional T-cells, PD-1 is upregulated upon T-cell receptor (TCR) mediated stimulation (45). Interaction with its ligands, PD-L1 and PD-L2, restricts further activation and proliferation of T-cells, thereby providing a central immune checkpoint to contain excessive immune responses (46, 47). This co-inhibitory signal is (at least partly) conveyed through downregulation of the PI3K pathway, providing direct antagonism to CD28-mediated costimulation (resulting in PI3K activation). On regulatory T-cells however, PD-1 seems to take on a distinctive role, which might be at least partly depending on the activity of CD28.

With an unaltered CD28 pathway, a conditional PD-1 knockout specifically in Tregs has been shown to enhance their suppressive capacity. In this context, Tregs lacking PD-1 out-proliferated conventional Tregs *in vitro*, protected NOD mice from diabetes and mitigated the severity of induced autoimmune encephalitis (48). In contrast, when the CD28-B7 interaction was disrupted (using CTLA4Ig) in transgenic mice overexpressing PD-1 on T-cells (including Tregs), PD-1^high^ Tregs demonstrated greater suppressive function, allowing for long-term survival of fully mismatched cardiac allografts (49). Transgenic PD-1^high^ Tregs under costimulation blockade expressed greater amounts of cytotoxic T-lymphocyte-associated protein 4 (CTLA4) and inducible T-cell costimulator (ICOS). Interestingly, active ICOS signaling in transgenic PD-1^high^ Tregs was required for the survival benefit in cardiac transplantation in this model. Although some of these differential results might also be explained by differences in the models used (auto vs. allo-immunity), these data indicate a complex interconnection between CD28, PD-1 and ICOS signaling in regulatory T-cells. In this context, our group has demonstrated that both PD-1 and CTLA4 are indispensable for maintaining chimerism and transplantation tolerance in a murine mixed chimerism model employing Treg-cell therapy and costimulation blockade with CTLA4Ig (50).

PD-1 upregulation has also been observed upon interleukin-2 (IL-2) stimulation of regulatory Tregs for *in vitro* and *in vivo* expansion. Asano and colleagues demonstrated an increased surface expression of PD-1 in Tregs during *in vitro* expansion in mice (with recombinant IL-2) and *in vivo* expansion using low-dose IL-2 in mice as well as human GvHD patients (51). Interestingly, when PD-1 signaling in Tregs was intercepted during expansion (in murine *in vitro* and *in-vivo* expansion) using PD-1 knockout Tregs or anti-PD-1 antibodies, Treg proliferation initially spiked, but then rapidly diminished due to FAS-dependent apoptosis induction and reduced BCL-2 expression on Tregs. These data indicate a central role for PD-1 as modulator of Treg homeostasis in clinically relevant Treg-expansion protocols.

Similar observations regarding the upregulation of PD-1 during *in vivo* Treg expansion have been made by our group using IL-2 complexes (IL-2 cplxs: IL-2 complexed with an anti-IL-2 antibody to sterically inhibit the binding to CD122 on CD8 T-cells and NK-cells while selectively expanding regulatory T-cells *via* CD25) for *in vivo* Treg expansion in a murine mixed chimerism model. In addition to PD-1, Tregs also upregulated ICOS and CTLA4 upon stimulation with IL-2 complexes (52). Together with CTLA4Ig these *in vivo* expanded Tregs facilitated long-term survival of fully mismatched cardiac allografts in mice (34).

The group of Robert Negrin has recently engineered an orthogonal interleukin-2 that selectively binds to an orthogonal IL-2 receptor (but not the native IL-2 receptor), that was introduced in regulatory T-cells (53). This model elegantly allows to provide IL-2 stimulation exclusively to transferred Tregs expressing the orthogonal IL-2 receptor. Also, in this context, stimulation with orthogonal IL-2 *in vitro* and *in vivo* was accompanied with an upregulation of ICOS in the transfected Tregs (PD-1 and CTLA4 were not assessed). Transferred orthogonal IL-2R Tregs facilitated induction of long-term mixed chimerism and subsequent donor-specific tolerance towards cardiac allografts.

These models, while using different strategies to deliver IL-2 selectively to regulatory T-cells, commonly demonstrate that IL-2 stimulation of Tregs is accompanied by an upregulation of PD-1, ICOS and CTLA4. In most of these reports and especially under costimulation blockade with CTLA4Ig, PD-1 upregulation was associated with enhanced suppressive function of regulatory T-cells. Another possible explanation for the observed disparity in Treg functionality with or without intact PD-1 signaling between autoimmunity and transplantation might be the PD-1 ligand (PD-L1) expression within the graft itself. PD-L1 is expressed by vascular endothelial cells and rapidly upregulated upon pro-inflammatory stimuli *via* interferon-gamma (IFNγ) and tumor necrosis factor alpha (TNF-α) (54). Reduced PD-L1 expression within cardiac allografts has been associated with an increased incidence of acute T-cell mediated rejections (55). Mechanistically, endothelial PD-L1 has been shown to reduce graft infiltration of CD8 T-cells expressing a memory phenotype (56, 57). Beyond this, endothelial PD-L1 might also interact with PD-1 on regulatory T-cells. A recent report suggests a novel role for PD-1 expressing Tregs in regulating endothelial trans-migration of lymphocytes through interaction with endothelial PD-L1 on lymphatic endothelial cells (58).

In this context, the effect that endothelial PD-L1 within the allograft itself has on PD-1 expressing regulatory T-cells could be of great interest and yet needs to be elucidated.

Also, on antigen-presenting cells, the PD-1 and CD28/CTLA4 pathways are strongly interconnected. Experimental data have suggested that CD80 (B7.1) and PD-L1 (CD274) can bind each other (59). The original assumption was that this interaction involves CD80 and PD-L1 expressed by two different cells (*trans*). Recent reports however suggest that CD80 and PD-L1 rather interact in a *cis* structure, forming CD80:PD-L1 heterodimers on the same cell (60). In this heterodimerized form, PD-L1 cannot be accessed by PD-1 on T-cells. This has been identified as one key mechanism by which PD-1 activity is restricted during T-cell activation to yield optimal T-cell responses (61).

Heterodimerization also impacts the accessibility of CD80 to its trans-ligands CD28 and CTLA4. While the binding of CD28 to CD80 is preserved, even in the cisCD80:PD-L1 form, CTLA4 cannot engage with heterodimerized CD80 [likely due to its multivalent zipper-like binding structure (62)]. Consequently, heterodimerized CD80 has been shown to be protected from CTLA4-mediated trans-endocytosis (63).

Thereby, upregulation of CD80 and increased CD80:PD-L1 heterodimerization on APC might lead to repression of co-inhibition by PD-1 (by reducing available PD-L1) and CTLA4 (by restraining trans-endocytosis of CD80) while preserving CD28 co-stimulation. In turn this might result in increased T-cell activation. Recent work by the group of Shimon Sakaguchi has demonstrated how regulatory T-cells can influence the balance between heterodimerized and “free” PD-L1 on APC (64): Through trogocytosis, Tregs can deplete (non-heterodimerized) CD80 from the APC’s surface, resulting in less cisCD80:PD-L1 heterodimerization and more “free” PD-L1 available to inhibit PD-1 expressing T-cells. These reports highlight the complex link between CTLA4-and PD1-mediated suppression of T-cell responses by regulatory T-cells.

**Figure 1:**
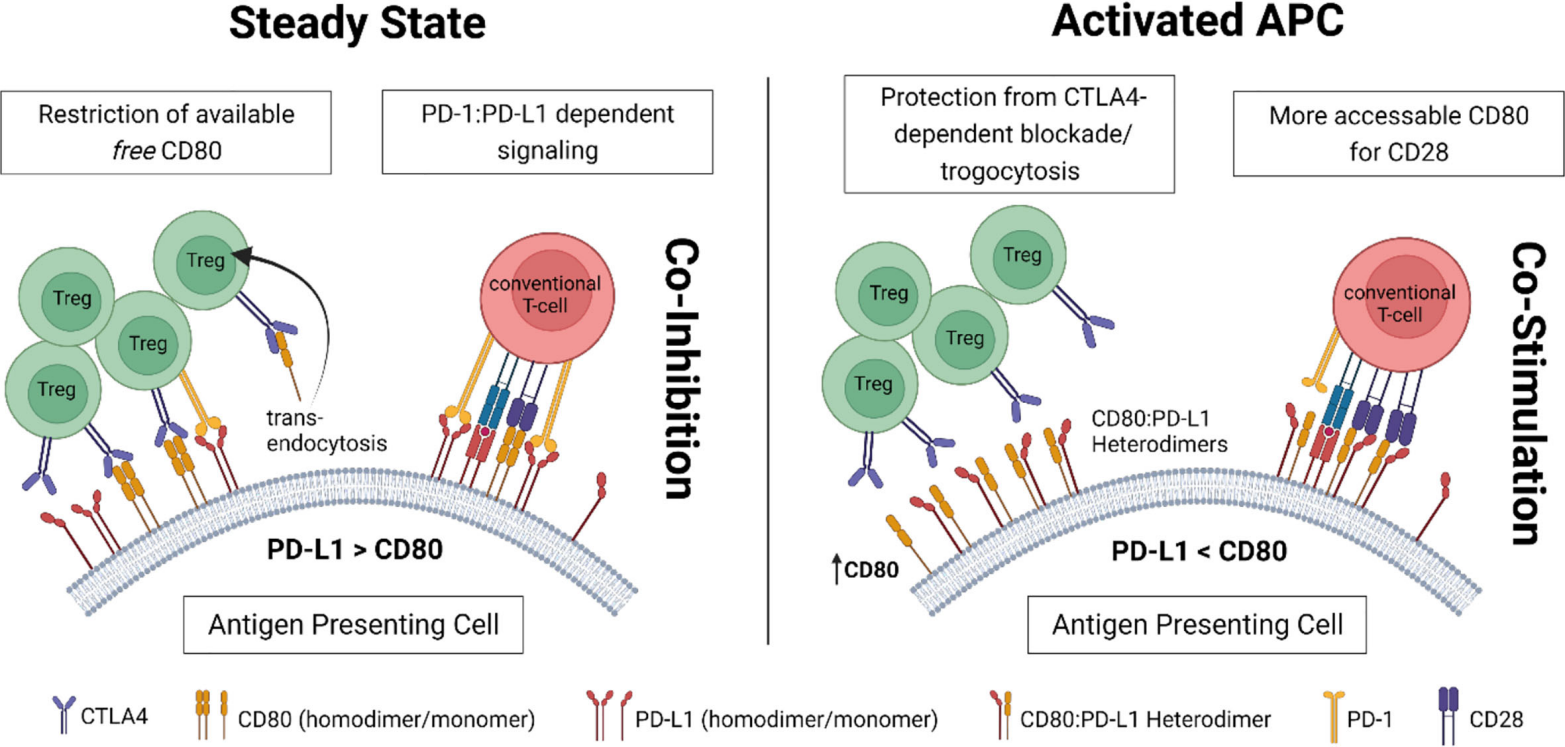
Proposed concept for the complex interconnection between Tregs and CD80:PD-L1 cis-heterodimers on antigen-presenting cells (APC).

Under steady-state conditions, Tregs control the amount of available CD80 on antigen-presenting cells (APC) *via* competitive inhibition and removal through trans-endocytosis. Both mechanisms rely on CTLA4 binding to CD80. This tight restriction on free CD80 results in limited formation of CD80:PD-L1 heterodimers and a high abundance of *free* PD-L1 (homodimers) providing co-inhibitory signals to engaging T-cells through PD-1 (**Figure 1**, left).

Under inflammatory conditions, upregulation of CD80 results in a higher rate of CD80:PD-L1 heterodimerization. While CD80 within those heterodimers is not accessible for CTLA4, it maintains affinity for CD28. Thus, in a heterodimerized form, CD80 can evade Treg-mediated control. As heterodimerized PD-L1, on the other side, loses affinity to its ligand PD-1 (on T-cells), in this setting, co-stimulatory signaling *via* CD28 prevails (**Figure 1**, right).

Cis-heterodimerization to PD-L1 might not only allow CD80 to evade Treg mediated control (via CTLA4), but potentially also costimulation blockade with CTLA4Ig. Experimental data investigating the effect of CTLA4-Ig (or other pharmacological interventions) on CD80:PDL1 heterodimer formation would therefore be of great interest to the field.

## CD28/CTLA4-B7 pathway and its blockade

The CD28/CTLA4 pathway is one of the most thoroughly studied costimulatory pathways. CD28 ligation *via* B7 molecules expressed on antigen presenting cells (signal 2) is crucial for T cell activation in combination with TCR/MHC interaction (signal 1). As absence of signal 2 in the presence of signal 1 renders T cells anergic (65) the concept of selective blockade of signal 2 has become attractive in order to therapeutically modulate immune responses in the clinical setting. Of note, CD28 engagement by B7 (CD80; CD86) is not only required for conventional T cells but also for Treg homeostasis (66, 67). Interestingly, CD86 appears to be the dominant ligand for Treg proliferation in spite of its approximately 10-times lower affinity to CD28 than CD80 (68). This can be explained by a constitutive high surface expression of CTLA4 on Tregs that selectively impair CD80/CD28 interaction (69).

In order to control conventional T cell activation, different approaches were used to inhibit B7/CD28 binding with the most promising strategy being the use of the fusion protein CTLA4Ig. Experimental research led to the development of abatacept, which now is approved for treating rheumatoid arthritis and recently also as GvHD prophylaxis by the FDA (70), and ultimately belatacept. Belatacept is a modified CTLA4Ig with a higher binding avidity to human B7 molecules (2-fold higher avidity for B7.1 and 4-fold higher avidity for B7.2 resulting in a 10 fold higher biological potency compared to conventional CTLA4Ig) (71), which has been approved for treating kidney transplant recipients. The main benefit of belatacept is probably the absence of nephrotoxic side effects compared to the conventionally used Calcineurin inhibitors (CNI) and in addition the improved patient adherence due to the monthly i.v. application of the drug. Long-term studies highlight the excellent allograft (kidney-) function which is preserved over time (72). However, in spite of the initial success, enthusiasm was dampened by higher rates of T-cell mediated rejections, especially in the early phase after transplantation, observed under belatacept compared to CNIs (73).

We have shown previously that the immunosuppressive capacity of CTLA4Ig is Treg-dependent at low but not high doses (74). However, the relationship between CTLA4Ig and Tregs remains incompletely understood. On the one hand there is a well-established negative impact of CTLA4/CD28-targeted costimulation blockade on Tregs (75, 76). Even though the exact mechanism of action has not been fully discovered it is likely that the negative effect on Treg numbers results from less available IL-2 (42) and decreased CD28 signaling which is essential for intrathymic Treg generation (67) and proper Treg function (77). This concept is further supported by the observation of a higher dependency on CD28 than conventional T cells (78). The negative effect of CTL4Ig on the number of Tregs is dose-independent and the main proportion of Tregs affected are helios^+^, nrp1^+^ tTregs (74). However, despite a reduction in Treg numbers *in vivo*, CTLA4Ig might also have a beneficial effect on Treg function and/or generation depending on the context (79).

For instance, murine iTreg generation and suppressor function was improved by CTLA4Ig *in vitro* (80). These findings are underlined by the observation that the addition of belatacept might enhance Treg mediated *in vitro* suppression of allogeneic immune response without affecting viability, proliferation or expression of functional Treg markers (81).

In a clinical study of kidney transplantation, there is a positive impact of costimulation blockade combined with mTor inhibition on Treg numbers with a sustained anti-donor suppressive activity compared to patients with a CNI-based immunosuppressive regimen (82). Similarly, belatacept treatment had no short or long-term effect on regulatory T-cell frequencies and *in vitro* functionality when compared to CNI in a *post hoc* analysis of the BENIFIT trials (83). Interestingly, there is evidence that costimulation blockade with CTLA4Ig might negatively affect CD44^high^ memory phenotype Tregs but not CD44^low^ naïve phenotype Tregs (84).

Some of the observed negative effects of CTLA4Ig on regulatory T-cells and the higher incidence of TCMR episodes might be attributed to the unintended interception of physiological CTLA-B7 binding. CTLA4 is upregulated on activated T-cells and delivers a co-inhibitory signal upon ligation with B7 (85). This co-inhibitory signaling is prohibited by CTLA4Ig. Directly targeting CD28 through non-crosslinking compounds might be a potential strategy to overcome this problem. Furthermore, CD28 blockade and CTLA4 on Tregs might synergize in their control over CD28 as they target the CD28-B7 interaction from two different angles. This has been shown experimentally by the group of Kathryn Wood in a humanized mouse model, where they demonstrated that direct CD28 blockade enhances Treg function and is superior to CTLA4Ig in prevention of allograft rejection (86). Two agents for direct CD28 blockade are currently under clinical evaluation in a phase I trial (NCT05238493) and a phase I/II trial in kidney transplantation (NCT04837092).

The effect of CTLA4 interaction with B7 molecules on APCs remains disputed. It has been suggested that CTLA4 might induce indolamine 2,3 Dioxygenase (IDO) *via* reverse signaling through B7 expressed on antigen-presenting cells (87). IDO is a tryptophan-catabolizing enzyme which leads to the production of pro-apoptotic metabolites (88). However, this concept has been challenged by the lack of IDO induction of CTLA4Ig in dendritic cells (89). The CTLA4Ig effect promoting chimerism in a murine model was also found to be independent of IDO (90). Moreover, the intracellular domains of CD80 and CD86 are short and due to their amino acid sequence are unlikely to transmit reverse signals (91). Notably, no IDO induction was detectable in liver transplant recipients treated with belatacept (92).

As Tregs constantly deplete B7 molecules from the surface of APCs, and CTLA4Ig reduces the numbers of Tregs, B7 expression on APCs is increased in mice under costimulation therapy compared to untreated animals.

**Figure 2:**
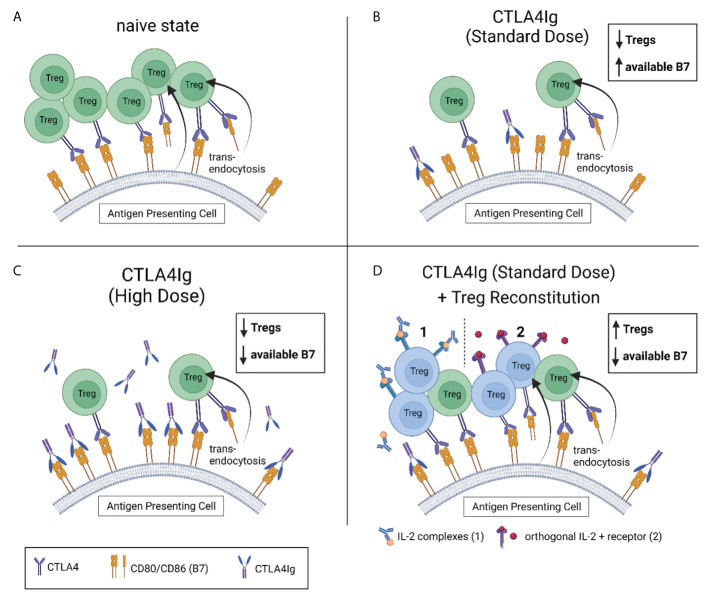
Strategies to compensate for reduced Treg levels under CTLA4Ig. **(A)** Tregs control the amount of available of CD80 and CD86 (B7) on antigen presenting cells (APC) through competitive inhibition and trans-endocytosis. **(B)** CTLA4Ig causes a dose-independent drop in Treg frequencies, contradicting their restriction on surface B7. Subsequently, more B7 is available for CD28-mediated co-stimulation. Standard doses of CTLA4Ig (10mg/kg) are not sufficient to bind all available B7. This can be compensated by administering higher doses of CTLA4Ig (50mg/kg) in the experimental setting **(C)**, or by reconstituting the recipient’s Treg levels (to or beyond naïve levels). Two promising strategies to reconstitute Tregs under costimulation blockade are depicted in **(D)** (1). Interleukin-2 complexed with an anti-IL-2 monoclonal antibody (IL-2 complexes) has been successfully used to *selectively* expand Tregs under costimulation blockade *in vivo*. (2) Engineered Tregs expressing a modified *orthogonal* IL-2 receptor that exclusively binds a modified (*orthogonal*) IL-2 have successfully been used for adoptive cell therapy in a mixed chimerism model. In both models, the re-established control of B7 expression on APCs by reconstituted Tregs has permitted sufficient immunosuppression with CTLA4Ig in standard dosing.

We suggested, that low dose CTLA4Ig might only insufficiently bind all available B7 receptors (93). Thus, there are two distinct strategies to overcome the resulting immune activation: 1) The administration of higher doses of CTLA4Ig (to bind all available B7 molecules (74); or 2) by increasing Treg numbers to ultimately decrease the number of B7 molecules expressed on APCs (34) (**Figure 2**).

Increasing Treg numbers by adoptive cell transfer of *in vitro* activated Tregs was insufficient in a mouse model of heart transplantation. Even though 3x10^6^ transferred Tregs were traceable for up to 16 weeks, we observed only a modest increase in Treg numbers that was absent in mice under costimulation blockade. We suggested that homeostatic control *via* the restricted availability of IL-2 might have limited the effect of adoptively transferred Tregs on overall Treg numbers.

Next, we aimed at increasing Treg numbers through IL-2 complexes (IL-2 cplxs). Thereby, we could successfully increase the number of regulatory T cells but also showed a synergistic effect of IL-2 cplxs and CTLA4Ig in reducing the expression of B7 molecules on dendritic cells (34).

However, there are further possible interpretations that can explain the observed beneficial effect of IL-2 cplxs on allograft survival under costimulation blockade. In a murine model of FoxP3 deficiency treatment with IL-2 cplxs can – at least partly - compensate the deleterious effect of the defective Treg compartment indicating other suppressive cells may be supported by IL-2 cplxs (3).

The close relationship between Tregs and CTLA4Ig is further underlined by the observation that in patients with DEF6 deficiency (an inherited syndrome characterized by immunodeficiency and systemic autoimmunity cause by an aberrant CTLA4 homeostasis) CTLA4Ig can improve the clinical phenotype (94).

## CD40-CD154 blockade

The interaction of CD40 on B cells and its ligand, CD154 (CD40L) is crucial for B-T cell crosstalk and activation. Consequently, great efforts have been taken to target this pathway therapeutically in transplantation and autoimmunity (95). In several models, blocking CD154 has shown to be superior compared to targeting CD40. This might be due to CD11b acting as alternative receptor for CD154, partially compensating for CD40 (96).

Blocking CD154 yielded promising pre-clinical results in several experimental models (97). However, clinical translation was hampered by thromboembolic complications during phase I testing (98). The originally developed monoclonal antibody targeting CD40L (hu5c8) caused immune complex-mediated platelet activation *via* FcγRIIa resulting in thromboembolic complications (NCT02273960). Recently, novel Fc-silenced constructs, devoid of any FcγRIIa binding have shown promising pre-clinical results (99).

Contrary to CTLA4Ig, blocking CD154 has been associated with an increase in Tregs across several murine (100–102) and non human primate models (99). Mechanistically, it seems that naïve CD4 FoxP3^-^ T cells are induced to become pTregs following transplantation under CD154 blockade (100). This effect might be one of many explanations for the observed synergy between CTLA4Ig and anti-CD154 in experimental transplant models (103).

## Outlook and future perspectives

Current challenges in transplant medicine including chronic allograft rejection and adverse side effects caused by conventional immunosuppressive regimens demand for novel strategies in order to further improve transplant outcome. Tregs are a powerful subset of immune cells that provide prompt and selective fine tuning of immune responses. The close association between costimulation blockade and Tregs observed in preclinical and clinical studies indicate a synergistical potential that merits further efforts in order to delineate the complex network between immune activation and regulation.

Several strategies are currently investigated in prospective trials including adoptive Treg transfer or Treg expansion by using IL-2 complexes. Also, new costimulation blockers are tested in preclinical and clinical studies. However, whether these efforts ultimately will result in reduced immunosuppression or even in donor-specific tolerance remains unclear.

## Author contributions

MM, CS, and TW jointly wrote and revised the manuscript. All authors contributed to the article and approved the submitted version.

## Funding

The authors’ work is supported by the Medical-Scientific Fund of the Major of Vienna (to MM, project number: 21050), the Vienna Science and Technology Fund (to TW, project number: LS18-031) and the Country of Lower Austria Danube Allergy Research Cluster (DARC) grant (to TW).

## Conflict of interest

The authors declare that the research was conducted in the absence of any commercial or financial relationships that could be construed as a potential conflict of interest.

## Publisher’s note

All claims expressed in this article are solely those of the authors and do not necessarily represent those of their affiliated organizations, or those of the publisher, the editors and the reviewers. Any product that may be evaluated in this article, or claim that may be made by its manufacturer, is not guaranteed or endorsed by the publisher.
